# Identifying and Diagnosing TDP-43 Neurodegenerative Diseases in Psychiatry

**DOI:** 10.1016/j.jagp.2023.08.017

**Published:** 2023-09-03

**Authors:** Simon Ducharme, Yolande Pijnenburg, Jonathan D. Rohrer, Edward Huey, Elizabeth Finger, Nadine Tatton

**Affiliations:** Department of Psychiatry (SD), Douglas Mental Health University Institute, McGill University, Montreal, Canada; McConnell Brain Imaging Centre, Montreal Neurological Institute, McGill University, Montreal, Canada; Alzheimer Center Amsterdam, Department of Neurology, Amsterdam Neuroscience (YP), Vrije Universiteit Amsterdam, Amsterdam UMC, Amsterdam, The Netherlands; Dementia Research Centre, Department of Neurodegenerative Disease (JDR), UCL Queen Square Institute of Neurology, London, UK; Taub Institute for Research on Alzheimer’s Disease and the Aging Brain, Department of Psychiatry (EH), Columbia University, New York, NY; London Health Sciences Centre Parkwood Institute (EF), London, ON, Canada; and the Alector, Inc. (NT), South San Francisco, CA.

**Keywords:** Frontotemporal dementia, neuropsychiatric symptoms, differential diagnosis, amyotrophic lateral sclerosis, limbic predominant age related, TDP-43 encephalopathy (LATE)

## Abstract

Neuropsychiatrie symptoms (NPS) are common manifestations of neurodegenerative disorders and are often early signs of those diseases. Among those neurodegenerative diseases, TDP-43 proteinopathies are an increasingly recognized cause of early neuropsychiatrie manifestations. TDP-43-related diseases include frontotemporal dementia (FTD), amyotrophic lateral sclerosis (ALS), and Limbic-Predominant Age-Related TDP-43 Encephalopathy (LATE). The majority of TDP-43-related diseases are sporadic, but a significant proportion is bereditary, with progranulin (GRN) mutations and C9orf72 repeat expansions as the most common genetic etiologies. Studies reveal that NPS can be the initial manifestation of those diseases or can complicate disease course, but there is a lack of awareness among clinicians about TDP-43-related diseases, which leads to common diagnostic mistakes or delays. There is also emerging evidence that TDP-43 accumulations could play a role in late-onset primary psychiatric disorders. In the absence of robust biomarkers for TDP-43, the diagnosis remains primarily based on clinical assessment and neuroimaging. Given the association with psychiatric symptoms, clinical psychiatrists have a key role in the early identification of patients with TDP-43-related diseases. This narrative review provides a comprehensive overview of the pathobiology of TDP-43, resulting clinical presentations, and associated neuropsychiatric manifestations to help guide clinical practice.

## INTRODUCTION

Neuropsychiatric symptoms (NPS) are common manifestations of neurodegenerative disorders, and are often early signs of those diseases.^1,2^ NPS of dementia include apathy, agitation, depression, delusions, and hallucinations, and frequently present to psychiatrists as late-onset (>40 years of age) behavioral change. The prevalence and nature of NPS vary according to dementia subtypes and the underlying neuropathology.^3^ In particular, NPS are highly prevalent in frontotemporal dementia (FTD), often preceding cognitive symptoms,^4,5^ and can lead to high rates of misdiagnosis, significant diagnostic delays, and may prevent patients from accessing appropriate resources.^6–9^ In this context, distinguishing FTD from primary psychiatric disorders (PPD) is particularly challenging, even among specialists, due to overlapping clinical presentations, heterogeneous clinical presentation, and the lack of molecular biomarkers for FTD.^6,10^ A deeper understanding of the relationship between NPS and the specific underlying neuropathology of FTD may improve diagnostic accuracy, disease management, clinical trial recruitment, and ultimately, access to emerging disease-specific therapies.

FTD is an umbrella term that includes the clinical syndromes of behavioral variant frontotemporal dementia (bvFTD), semantic variant primary progressive aphasia (svPPA), and nonfluent variant PPA (nfvPPA).^11,12^ The P ID-related disorders of amyotrophic lateral sclerosis (ALS) and the Parkinsonian-like syndromes of progressive supranuclear palsy (PSP) and corticobasal syndrome (CBS) can appear with different combinations of behavioral, cognitive, and language deficits.^13–15^ At the pathological levels, these syndromes are secondary to frontotemporal lobar degeneration (FTLD), which is a comprehensive term for progressive neurodegenerative changes largely in the frontal and temporal lobes. Among FTLD entities, pathological inclusions of transactive response DNA-binding protein 43 (TDP-43) can be seen in at least half of bvFTD cases, with significant neurodegeneration and atrophy of the frontal and temporal lobes as well as in subcortical structures.^16,17^ Up to 98% of all ALS cases have TDP-43 pathology,^18^ and TDP-43 pathological inclusions are reported postmortem in other neurodegenerative diseases that can present with NPS.^19,20^ As the common denominator across a wide range of neurodegenerative diseases, interest has increased in TDP-43 proteinopathies and how they manifest as NPS, with emerging evidence suggesting that TDP-43 may contribute to severe mental illnesses.^21^ Increasing awareness of FTD disorders and other TDP-43 proteinopathies among psychiatrists is critical to improving clinical recognition of TDP-43-related diseases among patients presenting with NPS.^22^ In this narrative review, we will 1) overview mechanisms of TDP-43 proteinopathy and clinical syndromes resulting from TDP-43 proteinopathy, 2) examine the prevalence of NPS among TDP-43 proteinopathies and genetic and molecular links to psychiatric disorders, 3) provide recommendations to improve the differential diagnosis of FTD vs PPD, and 4) discuss treatments in development for TDP-43 proteinopathies.

### Normal Structure and Function of TDP-43

TDP-43 is a heterogeneous nuclear ribonucleoprotein containing 414 amino adds encoded by the *TARDBP* gene. TDP-43 is comprised of an N-terminal domain with a nuclear localization signal, 2 highly conserved RNA recognition motifs (RRM1 and RRM2) that permit sequence-specific binding to RNA, and a glycine-rich, intrinsically disordered C-terminal domain whose cleaved fragments contribute to prion-like aggregation in vulnerable cells in the disease state.^23^ These TDP-43 structural domains mediate its binding to DNA, RNA, and proteins, including other TDP-43 proteins.^24–26^ While TDP-43 predominantly resides in the nucleus, it continuously shuttles between the nucleus and the cytoplasm to perform diverse functions in both compartments.^27^ TDP-43 regulates several aspects of RNA metabolism, including transcription, mRNA splicing, and translation.^25,28^ Cellular levels of TDP-43 are tightly controlled through a negative feedback mechanism in which TDP-43 regulates production of its own mRNA.^29^ TDP-43 is also involved in the formation of stress granules, which are cytoplasmic assemblies of translationally stalled mRNAs, translation initiation factors, and RNA-binding proteins formed in response to various cellular stressors.^30^ TDP-43 contributes to normal, noncell-autonomous physiological functions of glia, while deletion of glial TDP-43 argues for cell-autonomous roles for TDP-43.^31^

## PATHOLOGY

The histopathological hallmark of TDP-43 proteinopathy is the mislocalization and accumulation of hyperphosphorylated, ubiquitinated, and N-terminally truncated TDP-43 in neurons and glial cells.^18,25^ C-terminal domain fragments of TDP-43 exit the nucleus to form TDP-43 cytoplasmic inclusions.^32^ TDP-43 histopathology is classified into subtypes A-E based on inclusion morphology and subcellular distribution,^33,34^ and each subtype is associated with particular causal gene mutations or clinical syndromes.^35,36^

### Genes Associated With TDP-43 Proteinopathies

Genetic mutations in *TARDBP*, which encodes TDP-43, were first found to be a cause of sporadic and familial ALS and FTD disorders, providing a direct link between TDP-43 and disease pathogenesis.^37,38^ However, *TARDBP* mutations are a rare cause of ALS and FTD, accounting for only 1% of ALS cases and <1% of FTD cases.^39^ TDP-43 is found in both genetic and sporadic FTD and ALS. Apolipoprotein E *ε*4 (*APOE4*), the most common genetic risk factor for AD, has an increased frequency of TDP-43 pathology,^40^ and reactive astrocytes carrying the *APOE4* risk allele have been reported in ALS.^41^ Glial cell inclusions of TDP-43 appear characteristic of most ALS cases and can be sporadic or associated with *C9orf72, TARDBP*, or optineurin *(OPTN)* mutations.^42^

### TDP-43 Pathogenesis

Multiple disease pathways have been proposed, and evidence supports both loss-of-function (LOF) and gain-of-toxic function TDP-43 disease mechanisms.^43,44^ Under disease conditions, TDP-43 mislocalizes from the nucleus to the cytoplasm, which is recognized as a critical event initiating the disease cascade.^18,45,46^ TDP-43 binding to RNA enables it to play a critical role in transcription regulation and stress granule formation, and to have both positive and negative effects on the transcription of key mRNAs, such as neurofilament light and progranulin, in motor neurons.^47^ As a component of RNA granules in neuronal dendrites, TDP-43 can regulate local translation critical for synaptic plasticity and cognitive function.^48^ Under stress conditions, TDP-43 can partition into stress granules along with RNA and other proteins, and a defective stress response in neurons may promote the conversion of stress granules into pathological TDP-43 inclusions.^25,49^

### Clinical Syndromes

#### FTD

BvFTD is the most common clinical presentation of FTD, representing approximately 50% to 60% of all FTD disorders.^50,51^ It features pronounced changes in personality, including social disinhibition, apathy, loss of empathy, and perseverative or compulsive behaviors. SvPPA is typified by difficulties in single word comprehension and impaired word-finding, while deficits in nfvPPA language production present as effortful speech and agrammatism.^11,12,13^ TDP-43 causes approximately half of all sporadic bvFTD cases, and therefore one cannot identify with a high degree of certainty which patients have TDP-43-vs tau-related diseases. At present, it is unclear if there is dominance of one proteinopathy vs another in terms of behavioral features such as apathy, loss of empathy, and socially inappropriate behaviors.^54,55^ The proportion of svPPA and right temporal variant FTD cases that are due to TDP-43 is as high as 90%,^20^ and therefore clinicians can be more certain of a TDP-43 disease in this context.^54,55^

**FIGURE 1.**
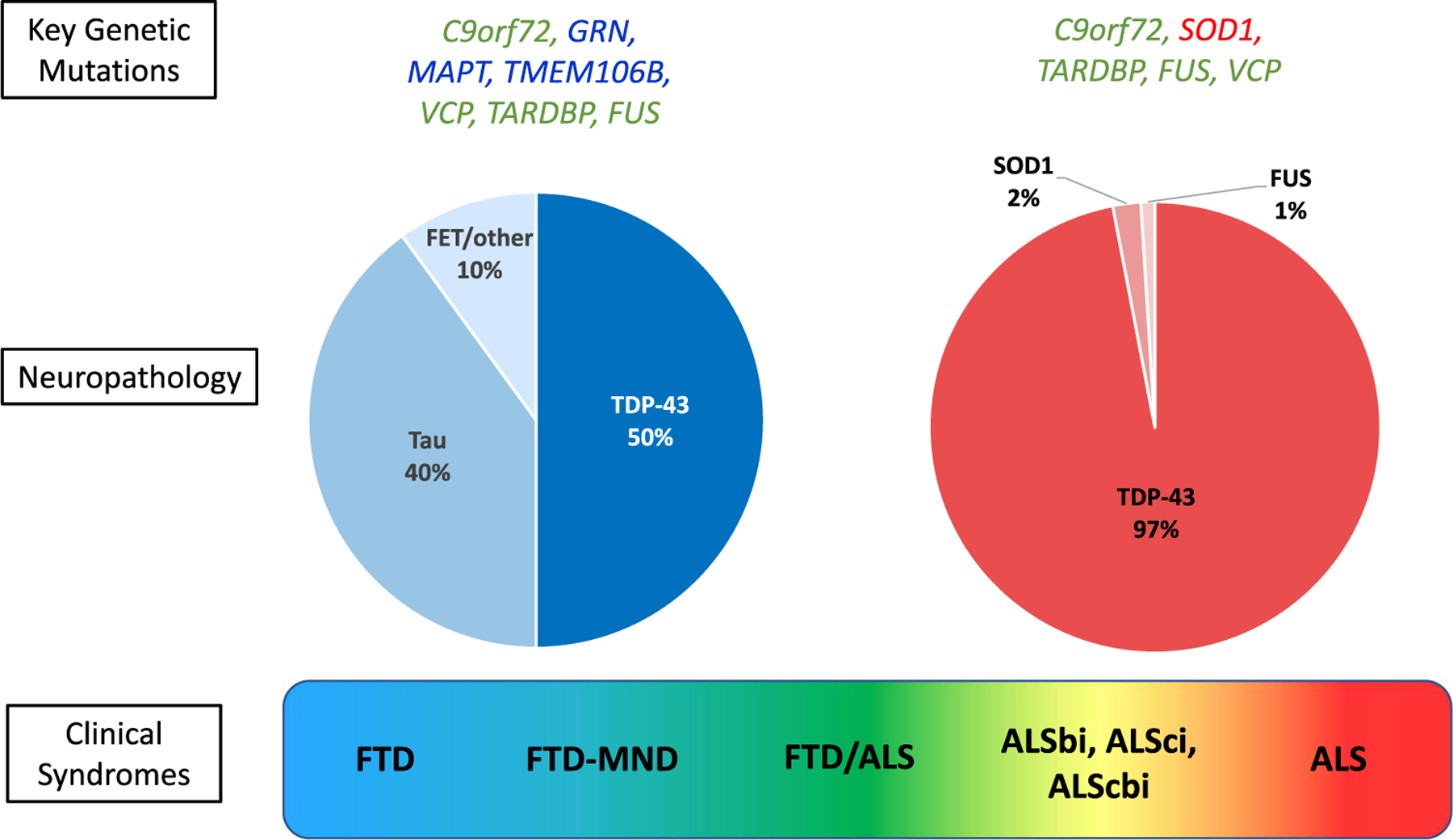
Pathological and genetic distribution in FTD and ALS. Visual representation of the continuum of clinical syndromes from FTD to ALS (bottom). The prevalence of pathological subtypes is depicted in pie charts with FTD on the left and ALS on the right. The most common genetic mutations are listed above the pie charts. ALS, amyotrophic lateral sclerosis; ALS-bi, ALS with behavioral impairment; ALS-cbi, ALS with combined cognitive and behavioral impairment; ALS-ci, ALS with cognitive impairment; FTD, frontotemporal dementia; MND, motor neuron disease.

Approximately 15% of patients with FTD show comorbid motor neuron disease (FTD-MND), while an additional 27% of patients show some evidence of motor dysfunction during the disease course^56^. Autosomal dominant gene mutations in progranulin (*GRN)*, microtubule associated protein tau (MAPI), and chromosome 9 open reading frame 72 (*C9orf72)* with hexanucleotide repeat expansions are the major heritable forms of FTD. FTD-*GRN* and FTD-*C9orf72* are associated with TDP-43 pathology, while FTD-*MAPT* is associated with tau pathology (Fig. 1).^57^

#### ALS

ALS is a progressive neurodegenerative disease affecting cortical motor neurons as well as lower motor neurons at the bulbar and spinal level; appearing first as muscle weakness, it eventually leads to muscle atrophy and respiratory failure.^58^ TDP-43-positive inclusions are present in most ALS cases (98%), with the exception of familial ALS resulting from gene mutations in superoxide dismutase 1 *(SOD1)* and fused in sarcoma *(FUS)*, which contain SOD1- or FUS-positive inclusion bodies, respectively.^59,60^ Most cases (90%−95%) of ALS are considered sporadic,^61,62^ and median age of onset is between 51 and 66 years.^63^ Approximately half of ALS patients develop deficits in executive function and behavior and can be classified by the revised ALS with FTD spectrum criteria.^64^ Typical ALS motor deficits can present with various combinations of mixed FTD behaviors and cognitive changes, or in some cases with nfvPPA language deficits. Recent structural imaging suggests that ALS-FTD with behavioral/cognitive involvement might be a phenotypic variant of ALS rather than a feature of worsening disease.^65,66^

### Limbic-Predominant Age-Related TDP-43 Encephalopathy

TDP-43 neuropathology is strongly associated with late-life cognitive decline^67^ in adults >80 years of age and is recognized as a unique disease entity named limbic-predominant age-related TDP-43 encephalopathy (LATE).^68^ LATE neuropathological change (LATE-NC) is associated with amnestic cognitive impairment mimicking Alzheimer’s disease (AD)^68^ and co-occurs with hippocampal sclerosis in ~40% of cases,^69^ AD neuropathology in 25%−37% of cases,^70,71^ or other mixed pathologies (e.g., Lewy body disease), and more rarely in isolation (~6%). A considerable proportion of cognitively normal older adults (11%−36%) have also been found to have TDP-43 proteinopathy, with increasing prevalence with older age.^21,72,73^ While LATE-NC is a unique disease entity for clinical and research purposes,^74^ clinical identification of LATE-NC is challenging in the absence of TDP-43 biomarkers.

### Others

Other primary TDP-43 proteinopathies include multisystem proteinopathy and the dementia Parkinsonism-ALS complex of Guam. Concomitant TDP-43 pathology has also been observed in AD,^75,76^ Parkinson’s disease (PD), dementia with Lewy bodies, Huntington’s disease, CBS, and PSP.^49^

## PSYCHIATRIC PRESENTATIONS IN TDP-43 PROTEINOPATHY

### FTD and FTLD

NPS are prevalent in FTD and are important to recognize in psychiatric practice as they may be the earliest manifestation of the disease. This prevalence has been derived from autopsy-confirmed cohorts (including both sporadic and genetic cases) and large genetic observational studies (only genetic cases). The frequency of psychotic symptoms (e.g., delusions and hallucinations) in neuropathologically confirmed FTLD ranges from 10% to 32%, with differences based on underlying neuropathology subtype, genetic variant, and clinical syndrome.^77,78^ Recently, Naasan et al.^79^ examined patterns of NPS in a large cohort of patients with autopsy-confirmed neurodegenerative pathology, including FTLD-TDP inclusions, FTLD-tau (including Pick’s disease, PSP, CBS), AD, and Lewy body disease. Patterns of NPS differed not only across major neuropathology types, but also by underlying FTLD-TDP subtypes. Patients with FTLD-TDP types A and B were more likely to have delusions (32%−35%) compared with patients with AD (16%) or FTLD-tau (2%−15%) pathology.^79^ Among patients with FTLD-TDP, psychosis was most prevalent among patients with FTLD-TDP type A (53%), followed by type B (42%) and type C (32%). Patients with FTLD-TDP types A and B more frequently reported hallucinations (24%−30%) than those with FTLD-TDP type C (5%). Patients with FTLD-TDP types A and B were more likely to have delusions in the early stages of disease (i.e., in the first 3 years after disease onset) compared with other diagnostic groups.^79^ In another study, patients with FTLD-TDP pathology were more likely to report paranoid and self-elevating delusions (e.g., grandiosity, erotomania) than those with any other pathology types. Another pathologically confirmed cohort study found that the presence of hallucinations was a differentiating clinical feature suggesting FTLD-TDP pathology, given that it was absent in FTLD-tau or FTLD-FUS pathology.^80^ This investigation also found that perseverative/compulsive behavior was significantly more prevalent in TDP-43 types B (93%) and C (77%) compared with other TDP-43 subtypes.^80^ Collectively, these studies suggest that NPS are particularly prevalent in TDP-43-related diseases, even in sporadic cases, and that the emergence of those symptoms in older adults should trigger an evaluation for other features of TDP-43-related diseases.

Longitudinal cohort studies on genetic FTD (such as the Genetic Frontotemporal Dementia Initiative [GENFI]) have revealed associations between the major FTD genetic variants and NPS across the disease trajectory. *C9orf72* expansion carriers typically show higher rates of NPS, including psychosis and somatic delusions, and more severe psychosis compared with noncarriers.^5,81–83^ The prevalence of psychotic symptoms among *C9orf72* mutation carriers ranges from 21% to 56%, and psychotic symptoms frequently precede behavioral and personality changes characteristic of bvFTD by up to 5 years.^5,78^ A similar finding of more frequent psychotic symptoms among *C9orf72* expansion carriers is also observed across the ALS-FTD continuum.^84^

While not as predominant as in *C9orf72* carriers, NPS are also common among *GRN* carriers. A GENFI cohort analysis found that the most frequent NPS among *GRN* carriers were depression and anxiety, particularly in the early (43%−56%) and late (40%−100%) stages of disease, compared with hallucinations (0%−32%) and delusions (0%−40%).^82^ Other studies of symptomatic *GRN* mutation carriers report similar rates of hallucinations (6%−25%) and delusions (6%−33%).^85–87^ Overall, *C9orf72* and *GRN* carriers appear to share more similar behavioral and NPS trajectories compared to *MAPT* carriers (i.e., the common FTD mutation associated with tau pathology), perhaps resulting from their shared TDF-43 pathology.^82^ These results suggest that while late-onset psychotic symptoms are most indicative of a TDP-43 proteinopathy, these patients can also present with significant anxiety and depressive symptoms. These variations in NPS profile across TDP-43 pathological subtypes might relate to differences in the affected neuronal network (e.g., more posterior parietal involvement in *GRN* carriers vs temporal poles for TDP-43 type C).^88^

### ALS

In addition to shared genetic and pathological features, ALS has significant clinical overlap with FTD, in what has been termed the frontotemporal spectrum disorder of ALS (ALS-FTD).^64^ Over 50% of patients with ALS exhibit some form of neuropsychological impairment based on the original Strong criteria.^64,89,90^ The recently revised Strong consensus criteria classify cognitive/behavioral impairment along a clinical spectrum, including ALS cognitively normal (ALS-cn), with cognitive impairment (ALS-d), with behavioral impairment (ALS-bi), with combined cognitive and behavioral impairment (ALS-cbi), and ALS-FTD.^64^ In a recent study, patients with ALS-bi, ALS-cbi, ALS-FTD, and bvFTD were all found to have similar patterns of NPS severity, supporting the ALS-FTD spectrum.^91^

A recent investigation of psychotic symptoms across the ALS-FTD spectrum revealed a high prevalence of psychotic features in patients with ALS (18%), ALS-cbi (22%), bvFTD (39%), and ALS-FTD (55%).^84^ Common symptoms across subtypes included thought broadcasting, thought repetition, and hallucinations.^92^ Further, across the ALS-FTD spectrum, *C9orj72* carriers had much higher rates of psychotic symptoms (63%) than noncarriers (22%). Collectively, these studies suggest that psychotic symptoms are common across the ALS-FTD spectrum and, while they are more common in patients with ALS-FTD, they may be under-recognized in ALS without associated cognitive impairment.

Interestingly, patients with ALS also have an increased risk of psychiatric disorders before an ALS diagnosis compared with controls.^93,94^ Using a large national record linkage database, hospitalization for a diagnosis of schizophrenia (SCZ), bipolar disorder (BD), depression, or anxiety was associated with a higher risk of subsequent diagnosis of ALS within the following year.^94^ This association weakened when the ALS diagnosis occurred more than a year after hospitalization, supporting the view that psychiatric symptoms are prodromal in ALS. Corroborating these findings, another nationwide registry study found an increased risk of psychiatric disorder preceding ALS diagnosis, which peaked in the year prior to diagnosis.^93^ These findings underscore the importance of evaluating patients with late-onset NPS for motor signs and symptoms, which can be prodromal manifestations of neurodegenerative disease.

### LATE

NPS have been reported in some patients with LATE-NC but do not appear to be a common feature. In one study using data from the National Alzheimer’s Coordinating Center (NACC), LATE-NC participants with severe impairment (i.e., Clinical Dementia Rating [CDR^®^] global score 2–3) were more likely to show symptoms of psychosis than FTLD-TDP participants with severe impairment.^74^ Among LATE-NC participants with severe impairment, the prevalence of visual hallucinations and delusions were 18% and 25%, respectively.^74^ LATE-NC has been compared more extensively to AD. Using the UK Brains for Dementia Research cohort, Liu et al.^95^ found that comorbid AD and LATE-NC was not associated with greater NPS burden than AD alone. Overall, it appears that clinically significant NPS can be a feature of advanced stage LATE; however, little is known about the role and prevalence of NPS in the prodromal stage of this disease.

## ROLE OF TDP-43 IN NONDEGENERATTVE MAJOR PSYCHIATRIC DISORDERS

Given the high prevalence of NPS in TDP-43-related disease, investigators have started exploring whether TDP-43 perturbations could also play a direct role in the context of major psychiatric disorders. Recent postmortem reports in psychiatry have noted TDP-43 neuronal inclusions in the hippocampus in a small set of BD cases^96^ and in patients with late-onset psychosis, including SCZ and BD.^97^ However, a prior study examining postmortem TDP-43 pathology in major psychiatric disorders observed no difference between older adults who had severe mental illness, primarily SCZ, and controls in the frequency, degree, or morphology of TDP-43 pathology.^21^ A postmortem investigation of TDP-43 proteinopathy in cognitively normal older adults also found no difference in NPS between adults with TDP-43 proteinopathy and those without.^73^

A larger body of work has investigated the genetic links between non-degenerative PPD and TDP-43 proteinopathies. Relatives of patients with ALS have an increased risk of psychiatric disorders, including SCZ (three-to fourfold higher risk), psychosis, suicide, and autism spectrum disorder.^93,98,99^ *C9orf72* expansion did not fully account for the increased risk of psychiatric disorders among ALS kindreds.^99^ Further, in a study of family members of patients with FTD and ALS, kindreds of *C9orf72* expansion carriers had higher rates of PPD, including SCZ and mood disorders, compared with relatives of *C9orf72* noncarriers.^100^ Although the precise genetic association remains unclear, these findings support the concept of a neuropsychiatric endophenotype in ALS/FTD kindreds.^99^

Of note, several association studies have explored *GRN* variability, plasma progranulin protein (PGRN) levels, and the risk for developing BD. In German and Italian cohorts, Galimberti et al^101,102^ have reported significantly lower plasma PGRN levels in patients with BD compared with controls, a finding which has been replicated.^103^ Medication was a potential confounder in BD patients, with the replication analysis showing that lithium influenced PGRN levels. Lithium-treated patients had significantly lower plasma PGRN levels compared with nonlithium-treated patients, although nonlithium-treated patients still showed significantly lower plasma PGRN levels compared with controls.^103^

At the mechanistic level, TDP-43 inclusions in astrocyte cytoplasm can activate astrocytes and induce inflammation and the secretion of pro-inflammatory factors that contribute to neurodegeneration^104^ and inflammation.^105^ In mood disorders, there is some postmortem evidence of abnormal glial pathology; young and mixed age group major depressive disorder patients appear to have less glial fibrillary acidic protein (GFAP) immunoreactive astrocyte density than control patients, while in late-onset depression there is increased density of GFAP immunoreactive astrocytes.^106^ Taken together, this evidence suggests that TDP-43 plays a critical role in glial cell homeostasis and glial regulation of neuronal function, which might theoretically contribute to psychiatric disturbances; however, neuropathological findings do not support a strong link between TDP-43 accumulation and major psychiatric syndromes.

## DIAGNOSIS OF DISEASES DUE TO TDP-43 IN PSYCHIATRY

### FTD

Patients with unidentified FTLD-TDP and LATE-NC can be encountered in general psychiatric practice. The most significant limitation in clinical practice is the absence of valid TDP-43-specific fluid or imaging biomarkers, except for genetic screening to determine if FTD is caused by *GRN, C9orj72*, or *MAPT* gene mutations.^10,107,108^ Therefore, diagnosis largely depends on the clinical assessment and a probabilistic method to identify likely TDP-43-related diseases.^6^

Clinical strategies for distinguishing bvFTD from PPD in cases of late-onset behavioral changes are similar for all pathological subtypes; however, the presence of delusions and hallucinations is particularly concerning for both the sporadic and genetic forms of TDP-43-related disease.^6,79^ Table 1 lists some of the red flags to look for during clinical assessment.

While there is consensus that neuroimaging should be performed in the diagnostic investigation of bvFTD, the sensitivity and specificity of standard magnetic resonance imaging (MRI) and positron emission tomography (PET) tracers for differential diagnosis of bvFTD vs PPD are insufficient in the early, ambiguous stages.^7,109^ Recent clinical practice recommendations to guide the differential diagnosis of bvFTD vs PPD suggest various approaches to improve diagnostic accuracy, such as the inclusion of at least 1 structured test of social cognition (e.g., Ekman 60 Faces Test, Social Cognition and Emotional Assessment [SEA], or Mini-SEA) to the standard neuropsychological battery for bvFTD,^6^ and increasingly, the use of neurofilament light chain as a differential marker between PPD and neurodegenerative diseases 110–112 In Edition, the Frontotemporal Dementia versus Primary Psychiatric Disorder (FTD versus PPD) Checklist is a recently developed bedside tool designed to help distinguish between bvFTD and PPD, although further validation is needed.^113^ While a significant proportion of patients with svPPA have NPS,^114^ the svPPA diagnosis is more straightforward as neuropsychological and speech assessments have strong utility in recognizing semantic deficits. Genetic testing may be warranted in any patient with unexplained late-onset behavioral disturbance (particularly psychosis) who has a first-degree relative with ALS or FTD.^6,115^

### LATE

LATE-NC presents in older patients with short-term recall deficits and is frequently mixed with other pathologies, sometimes accompanied by NPS-like psychosis as well as hippocampal sclerosis on MRI. Clinically, it is nearly impossible to accurately identify patients with LATE-NC as opposed to AD or mixed dementia. When performed, the absence of amyloid on molecular markers in an elderly patient with short-term memory deficits can be a due to the presence of LATE-NC changes; however, the prevalence of amyloid plaques in older adults in the age range of LATE-NC is very high.

### TDP-43 Biomarkers

Efforts are underway to develop sensitive fluid and neuroimaging biomarkers for FTD and TDP-43 proteinopathy. Until reliable FTD biomarkers can be validated and implemented in the clinic, it is important to use all available tools to increase diagnostic accuracy in patients with late-onset behavioral changes. Accurate and early diagnosis of FTD is paramount to facilitate appropriate treatment and enable ongoing and future clinical trials of investigative disease-modifying therapies.

## TREATMENT DEVELOPMENTS FOR TDP-43 PROTEINOPATHIES

Investigative therapies specific to FTD and ALS are being vigorously pursued to address the urgent unmet need for treatments. Currently, there are no FDA-approved treatments for FTD. Managing NPS in FTD is limited to nonpharmacological interventions (e.g., behavioral programs)^116,117^ and off-label psychiatric medications (e.g., selective serotonin reuptake inhibitors [SSRIs]), although evidence supporting the efficacy of these medications in FTD is minimal and certain medications, such as antipsychotics, carry an increased risk of adverse effects for elderly patients with dementia-related psychosis.^118,119^ Edaravone and riluzole are approved for use in ALS and demonstrate modest increases in survival, while the combination drug sodium phenylbutyrate/taurursodiol appears to confer a larger survival benefit by comparison.^120^ Novel drug candidates targeting *C9orf72* expansions and PGEN haploinsuffidency are in various stages of clinical trial development (see Boxer et al^121^ for review). The 2 greatest challenges of conducting clinical trials on these therapeutics in FTD are clinical trial recruitment and disease heterogeneity.^121^

### Therapeutic Strategies for TDP-43

It is unclear whether targeting a specific aspect of TDP-43 pathogenesis, such as cytoplasmic mislocalization, post-translational modification, or aggregation, will offer the greatest therapeutic potential.^122^ Several antibody-based interventions directly targeting TDP-43 are being investigated in cell lines and animal models of TDP-43 proteinopathy.^123^ Recently, a full-length monoclonal antibody targeting the RR1 domain of TDP-43 was shown to bind specifically to cytoplasmic TDP-43 in the brain and spinal cord tissues of postmortem FTD/ALS patients, and to reduce cytoplasmic TDP-43 in murine spinal cord neurons.^124^ The translational feasibility of these compounds has yet to be determined.

### Therapeutic Strategies for FTD-*GRN*

**FIGURE 2.**
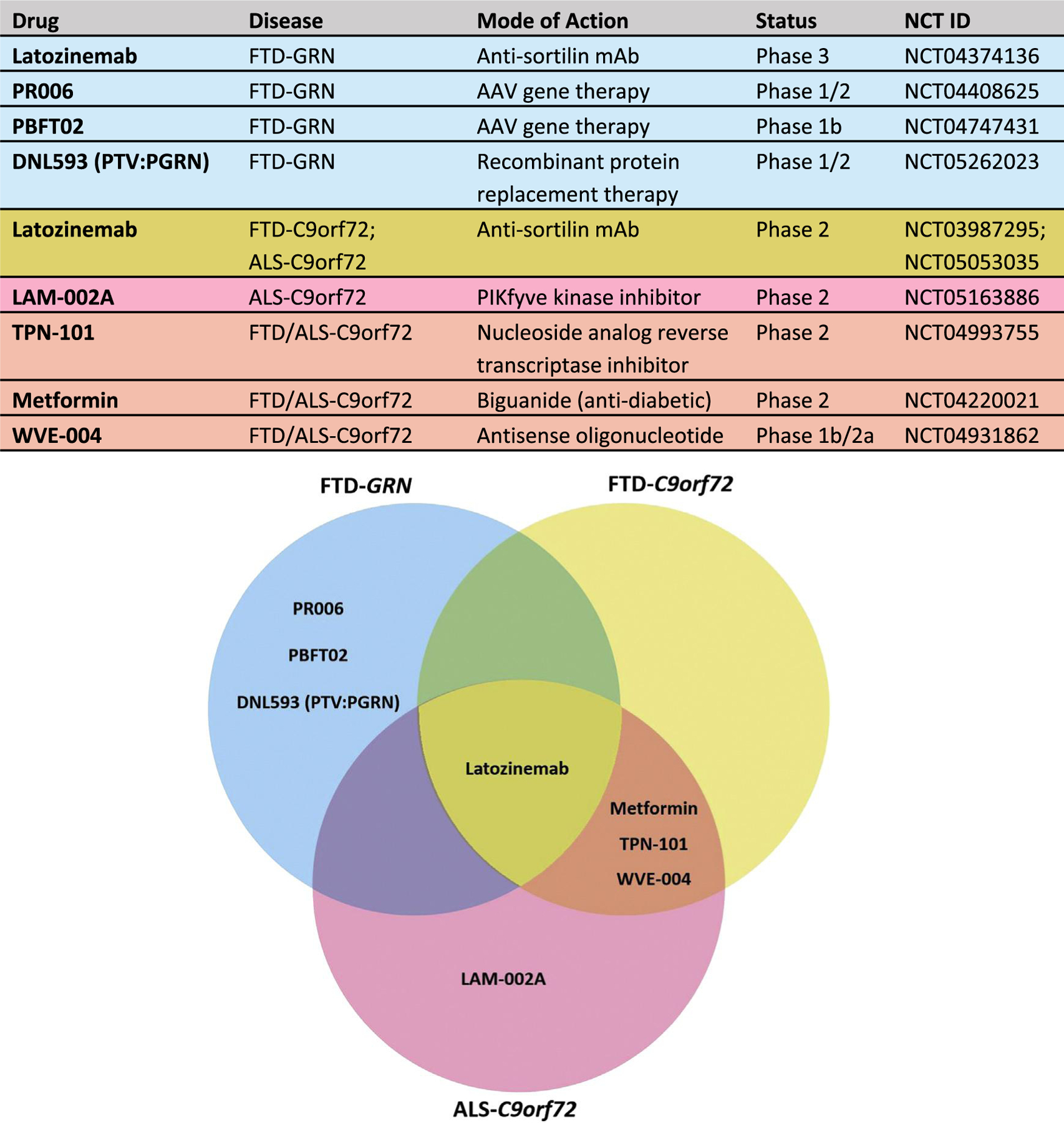
Clinical trials for FTD-*GRN* and FTD)/*ALS-C9orf72*. The table lists the drug, target disease, mechanism of action, status, and registration number. The Venn diagram at the bottom provides a visual representation of the drug distributions based on target disease and mutation. AAV, adeno-associated virus; ALS, amyotrophic lateral sclerosis; FTD, frontotemporal dementia; mAb, monoclonal antibody.

A growing body of evidence indicates that restoring PGRN may be an effective therapeutic strategy for FTD-GRN as well as other neurodegenerative diseases (reviewed elsewhere^125^). In addition to the PGRN haploinsuffidency disease mechanism in FTD-GRN, PGRN has neuroprotective and neurotrophic properties,^126,128^ and PGRN deficiency is a common feature of neurodegenerative diseases.^125^ Several approaches are in preclinical and clinical development to augment PGRN levels in FTD-*GRN*, including blocking the degradation pathway of PGRN, protein replacement therapy, gene therapy, and small molecule histone deacetylase (HDAC) inhibitors (Fig. 2).^125^

In the first approach, a monoclonal antisortilin human antibody, latozinemab (formerly AL001), was developed to block the sortilin-PGRN interaction and prevent PGRN degradation while retaining the ability of PGRN to have functional interactions through alternate trafficking pathways.^129^ The completed phase 1 study showed that latozinemab increased plasma and CSF PGRN to healthy control levels in GRN mutation carriers.^130^ Preliminary 12-month results from the phase 2 open-label study (NCT03987295) in patients with FTD-*GRN* suggest that latozinemab treatment improves multiple biomarkers of disease activity and may slow clinical progression relative to a GENFI2-matched control cohort as measured by the CDR^®^ plus National Alzheimer’s Coordinating Center FTLD Behavior and Language Domains Sum of Boxes (CDR^®^ plus NACC FTLD-SB) scale.^131^ A pivotal phase 3 trial for latozinemab is ongoing to evaluate its safety and efficacy in patients with FTD-*GRN* (NCT04374136).

Gene therapy has shown promise for treating rare monogenic diseases and may offer another strategy for elevating PGRN levels in patients with *GRN* LOF mutations. In this approach, DNA encoding *GRN* is delivered through an adeno-assodated virus (AAV) vector into the dstema magna. AAV-mediated *GRN* gene delivery has demonstrated proof of concept in murine models of PGRN deficiency,^132,133^ although safety concerns over *GRN* overexpression in the central nervous system were raised when a different AAV vector and intraventricular gene delivery were assodated with hippocampal neurodegeneration in one study.^134^ Translational safety and feasibility of AAV-mediated *GRN* gene therapy was demonstrated in nonhuman primates,^133^ and phase 1/2 trials are underway evaluating 2 different AAV vectors in patients with FTD-*GRN* (NCT04408625; NCT04747431).

Another therapeutic strategy under investigation for FTD-*GRN* is intravenous PGRN protein replacement therapy, in which recombinant PGRN protein is fused to an engineered antibody segment that binds to the transferrin receptor to increase blood-brain barrier transport and brain penetrance. A phase 1/2 trial is ongoing for PTV:PGRN (NCT05262023), a protein transport vehicle fused to PGRN, Lastly, HDAC inhibition was shown to enhance PGRN transcription in preclinical studies, although a phase 2 trial of a similar HDAC inhibitor did not find an increase in PGRN levels in participants with prodromal-to-moderate FTD-*GRN*.^137^

### Therapeutic Strategies for FTD- and ALS-*C9orf72*

Therapeutic strategies for treating FTD- and ALS-*C9orf72* have primarily focused on immunotherapy, antibody-based interventions, and gene therapy approaches (eg., RNA interference, CRISPR-based genome editing, AAV-mediated gene silencing, and AAV-mediated gene delivery including trophic factors).^123,138,139^ One strategy to counteract the gain-of-toxic function disease mechanism uses antisense oligonucleotides (ASOs) to selectively target repeat-containing RNAs for degradation while preserving *CSorf/2* mRNA levels. ASO-mediated therapy has demonstrated proof of concept in reducing nuclear RNA fod and dipeptide repeat proteins in preclinical studies.^140,141^ Recently, a phase 1 clinical trial of the ASO BIIB078 (NCT03626012) for ALS-*C9orf72* patiente did not demonstrate clinical benefit and the open-label extension was terminated (NCT04288856). Unfortunately, a phase lb/2a trial of another ASO targeting the *C9orf72* expansion transcript in FTD and ALS, WVE-004, was recently canceled due to lack of clinical benefit despite target engagement (NCT04931862).

Several additional clinical trials are studying new and pre-existing drugs in FTD*/ALS-C9orf72*. An open-label phase 2 trial is evaluating the safety, pharmacokinetics, and pharmacodynamics of latozinemab, an anti-sortilin antibody designed to block degradation of PGRN, in patients with *FTD-C9orf72* (NCT03987295). PGRN overexpression has been shown to reduce TDP-43 aggregation and improve survival in a mouse model of TDP-43 proteinopathy.^142^ Additional phase 2 trials are investigating pre-existing drugs in FTD*/*ALS-*C9orf72*, including: the widely-used antidiabetic agent metformin (NCT04220021; ALS-*C9orf72)*; TPN-101, a nucleoside analog reverse transcriptase inhibitor originally developed for the treatment of HIV (NCT04993755; FTD/ALS-*C9orf72)*; and LAM-002A, a PIKfyve kinase inhibitor that activates transcription factor EB and has been investigated in Thl 7-mediated inflammatory diseases (e.g., psoriasis) as well as B-cell non-Hodgkin lymphoma (NCT05163886; ALS-*C9orf72*).^143^

## CONCLUSIONS

Late-onset behavioral changes are frequently encountered in general and geriatric psychiatry and present a challenging differential diagnosis. TDP-43 proteinopathies such as FTD and LATE-NC can cause NPS in some patients and pose distinct diagnostic challenges due to symptomatic overlap with PPD, heterogeneous clinical presentation, and the lack of reliable biomarkers. Although much remains to be elucidated, the genetic, neuropathological, and clinical associations reviewed here suggest that NPS are related to the underlying neuropathology and could provide cues for better diagnostic recognition. Indeed, the presence of late-onset NPS, in particular psychosis, can be the initial manifestation of both sporadic and genetic TDP-43-related diseases, and psychiatrists play a key role in the identification and investigations of those patients. While an exploration of the prognostic significance of NPS in TDP-43-related disease is still needed, NPS are associated with faster disease progression and earlier death in patients with AD.^144,146^ Greater awareness of FTD and other TDP-43 proteinopathies is needed to further refine diagnostic recognition, prevent the use of ineffective treatments with potential negative side effects, and identify carriers of disease-causing mutations who may be eligible for clinical trials of investigational drugs or other appropriate treatment modalities. Improving diagnostic identification among psychiatrists could greatly contribute to the recruitment of eligible participants for research and enable adequately powered clinical trials, thereby accelerating the availability of disease-modifying therapies for TDP-43 proteinopathies.

## Figures and Tables

**TABLE 1.** Factors Suggestive of TDP-43 Proteinopathies in Patients With Late-onset NPS and Clinical Action Points

Red Flags for Possible TDP-43 Proteinopathy	Clinical Action Points
Late-onset hallucinations and/or delusions	Explore other indicators, cognitive testing, neuroimaging
Nonpsychotic late-onset NPS	Explore other indicators – investigate only if other factors present
Family history of FTD, ALS, or early-onset dementia	Collateral history from family +/− genetic testing and referral to genetic counselor if positive cases
bvFTD features, including hyperorality and stereotypies	Referral to specialty clinics, neuroimaging, neuropsychology, social cognition tests
Language disturbances	Neuropsychological +/− speech therapy assessment, neuroimaging
Cognitive deficits	Neuropsychological testing
Motor weakness	Neurological exam +/− EMG

*Notes:* ALS: amyotrophic lateral sclerosis; bvFTD: behavioral variant frontotemporal dementia; EMG: electromyography; FTD: frontotemporal dementia; NPS: neuropsychiatrie symptoms; TDP-43: transactive response DNA-binding protein 43.

## Data Availability

The data has not been previously presented orally or by poster at scientific meetings.

## Highlights

### • What is the primary question addressed by this study?

Neuropsychiatrie symptoms are often early signs of neurodegenerative diseases, contributing to misdiagnosis of these conditions. Among neurodegenerative diseases, TDP-43 proteinopathies are a common cause of neuropsychiatric symptoms and are under-recognized by clinicians, including geriatric psychiatrists. A better understanding of the relationship between neuropsychiatric symptoms and underlying TDP-43 neuropathology may improve recognition, diagnosis, and disease management.

### • What is the main finding of this study?

Psychiatric symptoms are prevalent in frontotemporal dementia (FTD) and related disorders and can vary based on TDP-43 neuropathology subtype. Depression and anxiety are often early presentations in FTD caused by *GRN* gene mutations, while FTD cases caused by repeat expansions in the *C9orf72* gene demonstrate a higher rate of psychosis and somatic delusions.

### • What is the meaning of the finding?

Late-onset neuropsychiatric symptoms can be the initial manifestation of sporadic and genetic TDP-43-related diseases, including FTD and amyotrophic lateral sclerosis (ALS), and TDP-43 pathology is often comorbid in Alzheimer’s disease. Psychiatrists must play a key role in the differential diagnosis of these patients, who require complex biopsychosocial interventions and may be eligible for emerging clinical trial opportunities for potentially disease-modifying therapies.
